# Hidden care: a qualitative exploration of the roles and responsibilities of language brokers

**DOI:** 10.1080/17482631.2024.2371536

**Published:** 2024-08-02

**Authors:** Renu Narchal, Rachel Hembrow

**Affiliations:** School of Psychology, Western Sydney University, Penrith, Australia

**Keywords:** Language brokering, family health and wellbeing, parent-child relationships, parent-child role reversal, migration, hidden care, informal care, young caregivers

## Abstract

This qualitative study explored language brokers’ conceptualizations of their roles and responsibilities within the family in Sydney, Australia. Semi-structured interviews were carried out to obtain retrospective accounts of language brokering experiences from 14 self-identified language brokers, 10 females and four males aged between 19 to 49 years (Mean age = 31) who started brokering between ages 7 to 16 (*M* = 11). Participants were from varied ethnic and socio-cultural backgrounds (Turkish, Lebanese, Filipino, Vietnamese, Chinese and Greek/German). Thematic analysis identified three primary themes: “Hidden Carers: When Parent-Child Roles are Reversed”; “Lost Childhoods: Personal Sacrifice for the Good of the Family”; and “Protecting Parents: Language Brokers as Custodians of Information.” Findings revealed that language brokering constitutes a form of hidden caregiving that carries with it several ramifications for the individual and their family. These findings have important implications for future research and policymakers working towards developing culturally responsive interventions that improve the health and wellbeing of language brokers and the resettlement experiences of migrants and their families.

## Introduction

The migration process poses complex challenges for new immigrants as they navigate the new host culture while maintaining their own cultural values (Zhang et al., 2020). The pace at which an individual adapts to the host country often depends upon their level of exposure to new social systems and their age upon arrival in the host country (Birman, 2006). Therefore, children adapt faster than other family members, leading to a parent-child acculturation gap (Birman, 2006; Guan et al., 2020). Within these migrant families, a culturally unique phenomenon known as language brokering often emerges as children act as linguistic and cultural mediators for their parents and other individuals from the broker’s own community including, members of their family, grandparents, neighbours, teachers, school peers to name a few (Angelelli, 2016; Tse, 1995). Language brokering can be thought of as the “natural translation” that bilingual individuals perform in everyday circumstances without any formal training (Harris, 1977). As a result of their cultural and linguistic knowledge, language brokers often play an essential role in their family and home functioning, as they help their parents navigate the new social systems of the host country (Tomasi & Narchal, 2020).

A growing number of the Australian population is not proficient in the English language. Approximately 820,000 individuals rate their English-speaking proficiency as “not well” or “not at all” in the 2016 census (Australian Bureau of Statistics [ABS], 2017). However, despite a large number of Australian migrants potentially relying on language brokers for interpretation and translation purposes, most of the findings within the Australian context are incidental, primarily focusing on the individuals whom language brokers interpret and translate for, rather than the brokers themselves (Clark et al., 2014; Pines et al., 2019; White et al., 2018). This has led to a call for more research on the experiences of language brokers within the Australian context (Tomasi & Narchal, 2020). Past research on language brokers has primarily adopted a benefit-detriment approach to uncover the impact this “non-normative” childhood experience has on individuals (Orellana & Phoenix, 2017). However, these studies often show mixed results, with many indicating that language brokering can have either a positive or negative impact on emotional wellbeing, cognitive development, personal relationships, and life experiences (Ceccoli, 2021; Kim et al., 2017; Morales & Wang, 2018; Tomasi & Narchal, 2020). Positive impacts on the young brokers for example include, feeling of greater empathy, maturity and self-esteem (Orellana & Phoenix, 2017) and feelings of pride in taking responsibility for the family (Arumí & Rubio-Carbonero, 2022; Ceccoli, 2022). The mixed and contrasting findings in the literature demonstrate the need to regard language brokering and its ramifications as a dynamic, multifaceted experience that is bound to a range of socio-cultural, individual, and family-related factors (Crafter & Iqbal, 2020; Dorner et al., 2008).

### Children adopting parental behaviors

Language brokering involves sophisticated linguistic and cultural adaptations to convey meaning between two or more adults. This adult-level responsibility may exceed the young language brokers’ cognitive, social, and emotional development (Tse, 1995). Other than interpreting and translating ongoing communication, language brokers must also respond to cultural backgrounds, power relationships, the ages and experiences of the speaker, and many broader contextual factors (Malsbary, 2013). Additionally, language brokers often play an essential role in helping their parents navigate and access financial, medical, and educational services, daily necessities, and overall family and home functioning (Hall & Sham, 2007; Weisskirch, 2012). Language brokers’ adoption of these responsibilities can cause instability within the family, as typical boundaries of parent-child relationships may become distorted (Oznobishin & Kurman, 2018).

However, whether language brokering has a significant impact on family dynamics and typical family structures is still largely debated (Weisskirch et al., 2020). Some researchers affirm that brokering leads to a form of “parentification” as child and adolescent language brokers assume developmentally inappropriate responsibilities (Arellano et al., 2018). Furthermore, family systems theory (Bowen, 1978) affirms that migrant parental reliance on brokers likely distorts normative hierarchical structures between brokers and their parents (Oznobishin & Kurman, 2018). Additionally, some quantitative studies have provided evidence of destabilized family structures through linking role-reversal or parentification to frequency of language brokering (Arellano et al., 2018). Concerningly, increased parentification scores in language brokers have been linked to increased depressive, anxiety, and somatic symptoms in addition to overall psychological distress (Arellano et al., 2018). However, language brokers’ own accounts of whether, and in what ways, they adopt adult-like roles and responsibilities remains largely unexplored.

In contrast to parentification, language brokering has been labelled a “family practice” (Crafter & Iqbal, 2021; Dorner et al., 2008). Comparisons have been drawn between brokering and other household chores or “pitching-in” tasks common within migrant families that originate from cultures that value the interdependence paradigm (Crafter et al., 2009; Dorner et al., 2008; Hall & Sham, 2007). Evidence comes in the form of language brokers’ perceptions and accounts of their interpreting and translating services (Dorner et al., 2008; Love & Buriel, 2007). Studies have shown that language brokers frequently report that interpreting and translating is just a “normal” part of their everyday lives (Crafter et al., 2009; Dorner et al., 2008). Further, studies have shown that parents and children work collaboratively while brokering, whereby parents remain active participants in the conversation (Ceccoli, 2019; Dorner et al., 2008; Love & Buriel, 2007). As a result, some researchers have theorized that language brokering extends this normative family dynamic rather than a parent relinquishing control of adult responsibilities (Dorner et al., 2008; Love & Buriel, 2007).

However, an issue with this framing is that research on young caregivers (such as those providing care for parents with disabilities or other health issues) reveals that children and adolescents frequently struggle to self-identify as carers (McDougall et al., 2018; Smyth et al., 2010; Stamatopoulos, 2018). In addition, Becker’s (2007) theories on informal care affirm that intra-familial bonds of love and reciprocity do not encourage individuals to view their caretaking behaviours as anything other than a “normal” part of their family relationship. Further, research on young carers from culturally and linguistically diverse communities has found that cultural expectations and complex parent-child relationships can make it particularly difficult for individuals from ethnic minorities and migrant backgrounds to identify their behaviours as a form of care (Hill, 2009). Thus, familial relationships and socio-cultural expectations relating to family obligation and reciprocation may be a barrier to language brokers recognizing their own care and support behaviours within the family domain (Bauer, 2015; Smyth et al., 2010). Therefore, framing the roles and responsibilities of language brokers as a type of family practice could contribute to language brokering experiences and outcomes remaining *invisible* and, or *hidden* within the family domain. However, more research is needed to understand how the roles and responsibilities of language brokers relate to “care work” (Becker, 2007).

## The current study

The language brokering literature is currently sparse, particularly from the Australian perspective, it is multidisciplinary, and has presented mixed and contrasting findings (Kim et al., 2017; Morales & Wang, 2018; Tomasi & Narchal, 2020). As a result, efforts to develop effective policies and interventions have been thwarted (Mier-Chairez et al., 2019). A cohesive framing of language brokering that considers the complex socio-cultural and intra-familial bonds tied to the act may result in an integrated and interdisciplinary understanding of this phenomenon that is seen widely across cultures and individuals. This, in turn, could have implications for dedicated support for language brokers and promote better settlement outcomes for migrants and their families (Mier-Chairez et al., 2019). Additionally, given the minority of research examining language brokers’ experiences in the Australian context (Tomasi & Narchal, 2020), this study will investigate the subjective experiences of language brokers currently living in Australia.

Further, much of the academic debate regarding language brokering surrounds the framing of the roles and responsibilities brokers perform and their positive or negative outcomes for the individual. By drawing on theories of informal care (Becker, 2007) and family systems (Bowen, 1978), this study aims to examine the roles and responsibilities of language brokers and to understand how they assist or hinder individual wellbeing, parent-child relationships, and family structures. Qualitative research that draws on existing theory is beneficial when attempting to understand data or observations that are not clearly understood due to conflicting results or interpretations (Collins & Stockton, 2018; Maxwell, 2013). Therefore, a qualitative methodology and semi-structured interviews will be utilized due to their ability to produce rich data that provides an in-depth examination of a particular social phenomenon (Mohajan, 2018). Accordingly, the current study seeks to understand:

1. In what ways do language brokers support, protect, and care for their parents while brokering?
2. How does a language broker’s adoption of adult-like roles and responsibilities relate to their parent-child relationships, family structure, and wellbeing?

## Materials and methods

### Design

Bowen’s (1978) family systems theory and Becker’s (2007) notions of informal care assisted in outlining the aims and goals of the study, developing interview questions, and informing methodological choices and the analytic approach (Collins & Stockton, 2018; Maxwell, 2013). Given the mixed results language brokering research has previously yielded, these existing theories were used to place a spotlight on data or observations that may have otherwise been overlooked or misinterpreted (Maxwell, 2013). In addition, a qualitative design involving semi-structured interviews was selected due to its ability to produce rich data that gives insight into the subjective experiences of participants (Mohajan, 2018).

### Participants

Fourteen bilingual individuals who self-identified as language brokers were purposely selected to participate in this study (Baker & Edwards, 2012). The inclusion criteria for this study required participants to be over 18 years of age; first or second-generation Australians; proficient in both English and their heritage language; and have experience interpreting and translating for at least one parent during childhood or adolescence and/or still language broker for them. Participants who consented to interviews were recruited from an online survey for a broader study on language brokers, Language brokers: The hidden figures. The researchers did not have any prior contact or relationship with the participants. They were recruited through flyers in the community. Snowball sampling (Goodman, 1961) was utilized, as it has proven to be a beneficial technique when a study is on a topic that requires insider knowledge to locate participants from within a hard-to-reach group (Streeton et al., 2004).

The interview sample comprised of 10 females and four males aged between 19 to 49 years (*M* = 31) from varied ethnic and socio-cultural backgrounds (Turkish, Lebanese, Filipino, Vietnamese, Chinese and Greek/German) who started brokering between ages 7 to 16 (*M* = 11). Individual participant characteristics presented in Table I indicate that brokering is not culture specific but carried out in immigrant families irrespective of their cultural heritage. Participants were assigned pseudonyms to maintain anonymity.

**Table I.** Demographic Characteristics of Participants.

Participant	Age	Gender	Ethnicity	Country of Birth	Languages Spoken ^a^	Age Started Brokering
Helen	42	Female	Lebanese	Lebanon	Arabic	7
Zara	37	Female	Lebanese	Lebanon	Arabic	11
Dina	43	Female	Lebanese	Australia	Arabic	8
Sara	22	Female	Lebanese	Australia	Arabic	10
Ayla	19	Female	Turkish	Australia	Turkish	16
Emily	49	Female	Lebanese	Australia	Arabic	15
Nadine	32	Female	Lebanese	Australia	Arabic	9
Nasir	27	Male	Afghan	Iran	Arabic	9
Lana	27	Female	Lebanese	Australia	Arabic	16
Ray	24	Male	Filipino	Philippines	Filipino	14
Ben	19	Male	Vietnamese	Australia	Vietnamese	11
Andrew	24	Male	Vietnamese	Australia	Vietnamese	12
Alexa	49	Female	Greek	Australia	Greek	10
Lily	21	Female	Chinese/German	Australia	Chinese/German	8

*N = 14*.

^a^Other than English.

### Ethical statement

Our study was approved by Western Sydney University’s Human Research Ethics Committee (approval no. H14368). All participants provided informed consent prior to engaging in the study.

### Procedure

The ethical considerations focused on ensuring confidentiality and anonymity for participants and gaining informed consent. Participants were made aware that they could stop the interview at any time or refuse to answer questions without penalty. Individual interviews were conducted online, using the video conferencing service, Zoom. Interviews lasted between 30 to 60 minutes. All participants were offered a $10 gift card to thank them for their participation.

The semi-structured interviews were deliberately open-ended to encourage participants to share their accounts of their language brokering experiences. The interview questions enabled participants to talk about: their experiences of providing protection, care, or support for parents while brokering; feelings about their parent-child relationship(s); and feelings about the impact brokering had on their family dynamics and functioning. Some examples of the interview questions include: “Tell me about a time when you have felt the need to protect or shield your parent(s) while brokering?” and “How would you describe the relationship that you have with your parent(s)?”

The interviews were audio-recorded using the online voice recording application Otter.ai. The auto-generated transcripts were checked and corrected for accuracy, and the verbatim transcripts were used for later thematic analysis. The excerpts that appear in the Results section were edited and a small number of irrelevant sentences and words have been replaced with ellipses (…), and excessive uses of colloquialisms, such as “like,” “you know,” and “um,” were removed. In addition, square brackets were used to de-identify participants, i.e., [treatment] was used instead of stating the specific medical intervention.

### Data analysis

Thematic analysis was conducted by implementing Braun and Clarke’s (2016) approach. The first step involved data-familiarization, transcribing data and re-reading transcriptions. During the second step, several first-order codes were generated and attached to the data, such as “withholding information,” “hiding own emotions,” and “reducing parental stress”.

In the third step, codes were searched for meaningful patterns or similarities to detect potential themes. At this stage, several potential themes were identified, including “Language Brokers as Protectors,” “Parent-Child Role-Reversal,” “Hidden Caregiving,” “Personal Sacrifice,” and “Withholding Information.” However, more development was needed to ensure themes considered the research questions and overall aims of this study. Thus, a more comprehensive thematic map was created during step four, whereby themes were re-examined and refined. During this step, it was found that the theme of “*Parent-Child Role-Reversal*” was too broad and, as a result, was unable to provide an in-depth understanding of participants’ experiences. In addition, it was found that aspects of this theme overlapped with other potential themes such as “*Hidden Caregiving*” and “*Personal Sacrifice*.” Therefore, the theme “*Parent-Child Role-Reversal*” was collapsed and combined with relevant aspects of the remaining potential themes. During this step, the potential themes’ Language Brokers as Protectors’ and “*Withholding Information*” were also combined due to many overlapping data extracts and codes.

The next phase of the analysis involved examining the three potential themes and writing a detailed description of each to understand the themes’ overall story. The names of the themes were chosen by the research team with great care to best demonstrate the complex experience of language brokering for the participants. They were adapted from quotes present in the dataset. For example, the theme: “Lost Childhoods: Personal Sacrifice for the Good of the Family” was adapted from a number of quotes presented in that section of the results. The label aimed to encapsulate the story of the dataset, whereby participants stated that they had “lost [their] childhood in a sense,” “had to grow up too quickly” or “mature faster” while maintaining that language brokering was a way to “give back” or “help the family.” The themes were then named “*Hidden Carers: When Parent-Child Roles are Reversed*,” “*Lost Childhoods: Personal Sacrifice for the Good of the Family*,” and “*Protecting Parents: Language brokers as Custodians of Information*.”

## Results

Three primary themes were developed from the data. These included “Hidden Carers: When Parent-Child Roles are Reversed,” “Lost Childhoods: Personal Sacrifice for the Good of the Family,” and “Protecting Parents: Language Brokers as Custodians of Information.” The primary themes reflect the participants’ experiences of language brokering and their commensurate roles and responsibilities within the family.

### Hidden carers: when parent-child roles are reversed

Participants provided accounts of performing emotional and instrumental caregiving behaviours to support their parents while brokering. However, most participants did not recognize their behaviour as a form of care and instead viewed language brokering through a normative framework of family obligation. Hence, this theme explores the idea that language brokers act as hidden carers as they frequently undertake caring roles that predominantly remain hidden and invisible within the family domain.

As language brokers, participants reported that they were required to assist their parents with “everyday hassles,” such as reading mail, paying bills, attending doctor’s appointments, and translating news articles. Participants would describe brokering in these instances as “helping my parents out.” (Sara, female, 22 years). However, it became evident that language brokering would frequently extend beyond translating and interpreting information for many participants. Several participants reported that parents would abdicate responsibility and instead “put the trust onto me” (Andrew, male, 25 years) when it came to making important decisions relating to the family. For example, Alexa (female, 49 years) stated that she was required to make decisions about “everything,” explaining:

They don’t really know processes and how it actually works. So, they relied on me a lot to make those decisions, and they just went with it. Like, “if that’s what Alexa says, then yes.” That’s basically all they do. (Alexa)

Similarly, Ray (male, 24 years) reported that his parents “couldn’t comprehend” the process of buying a home in Australia. As a result, Ray had to “take over”:

I just had to make the decision for them. I’d have to decide on an offer and then put it in for them. I mean, that’s a pretty big deal because obviously, it’s a lot of money. But when it came to the market and prices, I had a better understanding. (Ray)

Further, some participants spoke about emotional caregiving, as they were often required to diffuse their parent’s stress and anxiety. For example, Alexa reflected that she would often have to “reassure” her parents that the decisions they were making were the “right thing to do.” In addition, Lily (female, 21 years) reported that language brokering was a way to “help mum de-load her stress.” However, this often shifted the responsibility onto the participants, with some reporting that they would “carry the stress” (Dina, female, 43 years) so their parents would not need to. Evidence of this was provided by Alexa, who explained that she would take on her parent’s problems as if they were her own:

I’m used to taking on a lot of their issues, which is a lot for me. It’s like taking someone else’s problems and lumping them on top because then you don’t just have your stuff … you’ve got your parent’s stuff as well. (Alexa)

Similarly, Helen (female, 42 years) reflected that unlike herself, her childhood friends “didn’t have to deal with the problems that their parents are going through.” Another aspect of this type of emotional caregiving was exemplified by Dina, who reported that she became the “go-to person of the family” as a result of language brokering:

If something happens with my parents, I feel like I get the phone calls from everyone. (…) and I feel like my brothers don’t do that. They don’t get those phone calls. They’re calling me. And then I feel like I’m the one that’s always explaining things to other people, and I feel like I have to take control. (Dina)

Despite all participants in this study acknowledging that language brokering was a “big responsibility,” most of the participants did not frame language brokering as a form of caregiving. Instead, participants viewed language brokering services as a “normal” extension of their parent-child relationship. For example, Andrew described language brokering as a “habitual kind of situation. I know that I’m going to translate things, and I’ll just do it right away.” A similar sentiment was expressed by Lily, who explained, “My mum always communicated in Chinese. So, when I had to translate for her, it was without even thinking. It was quite normal to me.”

Only two participants (Alexa and Helen) positioned brokering was similar to a “carer’s role.” However, these participants emphasized that supporting and helping parents is “just something you do.” (Helen). Both participants compared it to the caregiving they perform for their own children. For example, Helen stated, “We get up as parents and make breakfast. We wash our children’s clothes. It’s the same kind of thing, but a bit in reverse.” Similarly, Alexa stated, “I feel like they’re my children (…) I treat them like my children.”

These feelings were often entwined with a participant’s ethnic identity and traditions. Participants stated it was “expected” of them to “protect” and “support” their parents. For example, Helen explained that “Middle Easterners have this sense of responsibility for our parents,” and the need to support them was a “culturally ingrained thing.” Alexa noted a similar practice within her own culture, stating, “Coming from a Greek background, the children look after their parents till the last breath.”

### Lost childhoods: personal sacrifice for the good of the family

Language brokering often comes at a psychological cost to the individual. Motivated by intra-familial bonds, ethnic identity and traditions, participants frequently reported sacrificing normative childhood experiences, psychological wellbeing, and personal goals or needs in order to help and support the family.

According to some participant accounts, sacrificing individual needs was seen as a way to fulfil parental expectations. For example, some participants reported that from a young age, their parents impressed upon them the notion that children “help the family” (Helen). Reflecting on high parental expectations, Dina recalled comments made by her father when she struggled to interpret accurately:

When it became technical, I didn’t know how to interpret (…) and my dad will get really frustrated like, “why do I send you to school? Why do you go to school? Do you go to school to help yourself? You don’t go to school to help us?”. (Dina)

In addition, language brokers in this study indicated a sense of “indebtedness” towards parents. For example, Andrew explained that brokering was “a way to give back to them for giving me this life.” This was also exemplified by Alexa, who explained that her parents had “gone without so that I could have.” Like Andrew, this instilled within Alexa a sense of obligation to “give back” to her parents no matter what through language brokering:

I have the utmost respect for my parents. I’ve seen how hard they’ve worked. I’ve seen what they’ve gone through (…) So, I think well, of course I’m going to look after them, and of course I’m going to do whatever is possible that I can do … of course I will. (Alexa)

In addition to obligation and reciprocation, love and close family relationships were also factors in participants’ decisions to interpret and translate for parents. For example, Emily (female, 49 years) stated that she and her siblings were motivated to help her parents because “we had the love and respect for them.” A similar sentiment was also expressed by Nadine (female, 32 years), who reflected that her relationship with her parents was “positive” and interpreting and translating for her parents “built a stronger connection because it felt like at one stage, I needed them in my life and then, later on, they needed me, which made the connection stronger.” These experiences highlight the role that intra-familial bonds of love, obligation, and reciprocity play in the brokers’ motivations to help and support their parents through language brokering.

However, for many participants, the notion of “helping the family” (Helen) and “giving back” (Andrew) encouraged participants to give their language brokering responsibilities priority above their individual needs and goals. This indicates that the same familial bonds that encourage participants to broker for parents may also foster an environment where language brokers cannot negotiate a reduction in their brokering responsibilities. For example, Emily expressed that since her parents are “always there for me,” language brokering was a way to “be there for them.” As a result, Emily explained that she became her parent’s “right hand” and began to “naturally” sacrifice spending time with friends and doing homework to be her mother’s interpreter and translator.

According to some participant accounts, the struggle to balance the dichotomies between their individual needs and their language brokering responsibilities continued into adulthood. Alexa exemplified this when stating, “you feel responsible for them. There’s a lot of times when you’re sort of torn between your family and your parents.” In addition, Alexa reflected that even though she would “love to go on an overseas trip with my family,” she would not because her non-availability for her parents would cause her “anxiety.” A similar sentiment was expressed by Dina, who struggled to balance her child-rearing and work-related responsibilities with her brokering obligations:

I feel like I’m going through my own stuff. I’m still trying to figure out this whole single mum thing. (…) he wants me to go to every appointment. And then I’ve got other things that I need to do, like I’ve been trying to manage work, study, and four kids on my own. (Dina)

Placing language brokering responsibilities above individual needs and goals had implications for participants’ wellbeing and development. For example, many participants in this study reported a general sense of “loss” when asked to reflect on their childhood experiences. Participants often stated that they were required to “grow up too quickly” (Alexa) and “mature faster” (Ben, male, 19 years) as a result of language brokering. Helen said that she had “lost her childhood in a sense” because of her brokering responsibilities. Helen explained that while her childhood friends were able to maintain “that sense of innocence,” her childhood offered no room for “sheltering” as brokering required her to “confront real-life situations from a young age.” In addition, some participants were able to recognize that the level of responsibility they had as children was not “typical,” as highlighted by Nasir (male, 27 years), who stated, “it isn’t really the norm for the average nine-year-old to take on so much responsibility.”

Additionally, some participants placed their language brokering responsibilities above their psychological wellbeing. Despite many participants finding language brokering “stressful,” “frustrating,” and “confusing,” they “hardly ever said no” (Dina). For example, Andrew reported that he would still translate and interpret for his parents even if he “wasn’t in a good place” mentally. This often resulted in Andrew having emotional outbursts stating, “I can blow up.” Andrew explained that he would frequently “get annoyed and be like, ‘why are you bugging me like this. Don’t bug me.” A similar sentiment was expressed by Dina, who stated that her “mental health had really suffered” and believed she had “developed anxiety” due to the stress of brokering for her parents. Dina reflected that she would often “wish I was just one of my brother’s” so she would not be the one to interpret or translate for her parents as frequently. This also highlights the gendered role of brokering and is further supported by the participants in this study where 10 participants were females and only 4 were males.

### Protecting parents: language brokers as custodians of information

Participants in this study were unanimous in reporting that while brokering distressing information or events, they attempted to shape or sanitize the nature of what was being communicated to “protect” their parents, acting as custodians of information. Participants seemed to adopt several strategies to shape the nature of the information they were brokering, including using humour, filtering information, distraction, and altering words. However, the most common technique used by participants was to “sugar-coat” (Lily) what was being said. For example, attempting to break the news in a “positive” or “reassuring” way. This was exemplified by Helen who described the experience of breaking the news that her mother had been diagnosed with cancer:

I had to be the one to sort of tell her that you’ve got cancer (…) It was really hard for me to do because I needed to sort of tell her in a way where it was more of a reassurance like “you’ve got cancer, but everything’s gonna be okay (…) I was like “mum, it’s the best cancer you can possibly get … they’re going to treat it, and I don’t want you to be afraid.” (…) I had to say it like that. I couldn’t tell her it’s metastasized. I wouldn’t even know the words to use for that in Arabic. (Helen)

When information was particularly distressing, participants frequently reported that they would also need to regulate their own emotions to ensure their parents remained unaware of the seriousness of a situation. For example, Lana (female, 27 years) explained that she moderates her feelings whilst brokering in stressful situations to not “leave an effect on her” mother. Alexa recounted a brokering experience where she needed to interpret distressing information relating to her mother’s health:

I was just calm, so try not to show my emotions so that they won’t reflect on them. Do you know what I’m saying? Like, because they were very much looking at the way I came across to see how bad the situation was. (Alexa)

However, according to some participant accounts, language brokers would go beyond “sugar-coating” and “filtering information” and withhold distressing information in its entirety. For example, Ray reported that he would often “have to lie” to his parents “because it’s better for them” and their “peace of mind.” Nasir also mentioned that to protect his mother, he and his siblings collectively decided to “avoid translating or telling her any information that might have been overwhelming or distressing to her.”

For some language brokers, their ability to gatekeep and influence certain information was to enable their parents to take decisions that had implications for them and their health. For example, Dina explained that when she interpreted and translated information relating to her father’s health issue, she often “buffed” the information, and her interpreting was “not so accurate.” Dina stated that her father was “very stubborn about not wanting to access [treatment]”:

We tried our best to convince him. Even though maybe it wasn’t so accurate. There are a lot of risks in any procedure (…) There are risks in everything, any type of surgery, even anesthetic. So, if I was to list all the risks, he would have freaked out (Dina)

This led to Dina and her brothers collectively deciding to “just tell him what we wanted him to know.” Dina said that this had a “huge effect” on her as she knew “exactly how inaccurate my interpretations were,” but she wanted her father to “hang around longer.” These experiences highlight the ways that language brokers can influence communication while brokering. Further, they demonstrate the implications of language brokering on parent-child dynamics and family structures.

## Discussion

The findings of this study demonstrate that ongoing language brokering responsibilities constitute a form of hidden caregiving that has several ramifications for the wellbeing of the individual and their family structures and relationships. Motivated by complex socio-cultural expectations and intra-familial bonds of love, obligation, and reciprocity, language brokers in this study were found to sacrifice their own psychological wellbeing, normative childhood experiences, and personal goals or needs to accomplish their language brokering responsibilities. Further, destabilization of family structures emerged in some instances as participants took on adult-like roles that distorted normative hierarchical systems within the family (Arellano et al., 2018; Bowen, 1978).

Participant accounts revealed that language brokers undertake a substantial and significant role that far exceeds normative expectations of familial help as compared to their non-language brokering counterparts. Children in immigrant families grow up with a greater sense of responsibility, and obligation for their families which continues well into adulthood and also for a lifetime for some (Del Torto, 2008). Further, brokers develop enhanced linguistic and social competence as they communicate in two languages and serve as go between adults negotiating with them and influencing content, emotions and attitudes of those they mediate for (Tse, 1995). Thus, the sociocultural aspects become dominant rather than the linguistic ones.

Findings indicate that parents often relied upon participants’ knowledge and experiences to access daily provisions, necessities, and services essential to family functioning (Becker, 2007; Daly & Lewis, 2000). However, language brokers in this study framed their roles and responsibilities through normative discourses of family connection and relationships (Becker, 2007; Bowen, 1978). This was evidenced by participants positioning their brokering responsibilities as a normal, routine part of their everyday life rather than a form of kinship care (Cline et al., 2011; Dorner et al., 2008; Love & Buriel, 2007). Thus, when individuals provide enduring care for family members, family bonds and connections prevent parties from viewing care behaviours as anything other than a “normal” family relationship (Daly & Lewis, 2000; García-Sánchez, 2018; O’Connor, 2007; Smyth et al., 2010). Therefore, it is not surprising that only few language brokers positioned themselves as caregivers, given the strong and complex intra-familial bonds evident in participants’ descriptions of their family relationships.

In addition to intra-familial bonds of love and respect, factors like complex socio-cultural expectations relating to ethnic identity and traditions posed additional barriers for language brokers in identifying their caregiving behaviours. Family or filial obligation appeared to play a significant role in language brokers’ motivation to interpret and translate for parents, as evidenced by participants’ positioning of language brokering as part of their duty of care as children (Weisskirch, 2012; Wu & Kim, 2009). Consistent with previous research (Buriel et al., 1998; Chao, 2006) brokering responsibility seemed to be placed more frequently on the daughters. Duty of care for the family is frequently depicted in collectivist cultures that value interdependence, mutual obligation, and prioritization of the family (Giacomin & Jordan, 2017; Weisskirch, 2012).

Further, it became evident that ethnic identity and family traditions play a role in keeping language brokering hidden within the family domain. For example, some participants recognized that their language brokering constituted a carers role and resulted in some adverse outcomes, such as increased psychological distress and missed opportunities in social and educational contexts (Arellano et al., 2018; Morales & Wang, 2018). However, cultural scripting made it difficult to view their complex parent-child relationships as anything other than expected (Dorner et al., 2008; Love & Buriel, 2007). This is particularly important as it may indicate that ethnic identity and traditions prevent or delay language brokers’ help-seeking behaviours, as has been the case for other ethnic minority and migrant communities (Magaard et al., 2017).

Children and adolescents, when placed in stressful situations acquire social and emotional competence at the cost of their psychological wellbeing (Hetherington, 1999). This was reflected in the current study as participants frequently reported increased maturity, independence, and enhanced decision-making skills while simultaneously acknowledging that language brokering had resulted in increased feelings of anxiety and psychological distress (Byng-Hall, 2008). While quantitative research has previously demonstrated that language brokering is linked to increased depressive and anxiety symptoms (Morales & Wang, 2018; Rainey et al., 2014), participant discussions surrounding their roles as language brokers revealed that they frequently place their language brokering responsibilities above their own psychological wellbeing. This was manifested in participants’ reluctance to refuse or decline to broker for parents, despite recognizing that they were doing so at the cost of their mental health and wellbeing. The intra-familial bonds and socio-cultural expectations motivate language brokers to take on enormous responsibility fostering an environment where language brokers cannot negotiate either with their parents or themselves, a reduction in their brokering responsibilities to improve upon their own wellbeing.

Further, participants sacrificing individual goals and personal needs to help and support the family was evident, which supports the existing literature (Aumann & Titzmann, 2019). This had implications on participants’ development and identity formation, as language brokers report that they were compelled to grow up too quickly and mature much faster than their age-matched peers. Additionally, participant accounts revealed that there was a general sense of loss in the context of their childhood experiences. Past literature affirms that informal caregiving activities performed by children and adolescents can cause a heightened sense of “missing out,” particularly in social and educational contexts (Stamatopoulos, 2018).

Indicative of disruption to normative hierarchical systems within the family (Bowen, 1978), the findings revealed that language brokers were placed in a position of power as custodians of information due to their ability to influence and moderate communication (Crafter & Iqbal, 2020). As evidenced by participants’ acknowledgement of withholding distressing news and events from parents, language brokers would frequently utilize their position of power gained through brokering responsibilities to protect parents as they deemed necessary (Orellana et al., 2003). This draws attention to an important issue surrounding informed consent when utilizing family members as informal interpreters and translators, specifically in medical and healthcare settings (Antonini et al., 2017; Goldsmith et al., 2008; Pines et al., 2019).

Further, the adoption of these roles had several implications for language brokers’ family dynamics. Participant accounts made it clear that, as children and adolescent language brokers, they felt a level of responsibility for their parent’s wellbeing, thus adopting strategies to reduce parental stress and discomfort. Participant accounts revealed that this could negatively affect their emotional wellbeing, as they were often compelled to regulate their own emotions and carry the stress of a problem so that their parents would not need to (Wu & Kim, 2009). However, the role-reversal present amongst language brokers and their parents did not have the detrimental impact on familial relationships that westernized notions on the “parentified” child would suggest (Arellano et al., 2018). Overall, participant accounts revealed that parent-child relationships were positive, including greater empathy and maturity (Orellana & Phoenix, 2017) evidenced by participant reports of language brokering strengthening parent-child bonds and contributing to closer familial relationships (Dorner et al., 2008; Love & Buriel, 2007; Wu & Kim, 2009). These findings demonstrate the importance of viewing language brokering through a culturally specific lens of interdependence (Dorner et al., 2008).

### Strengths and limitations

A strength of this study was the use of existing theory and qualitative methodology to thoroughly examine the roles and responsibilities of language brokers. This produced a rich data set that provided insight into language brokers’ own conceptualizations of their brokering experiences and how it affected their wellbeing, parent-child relationships, and family structures. Combined with existing theory (Becker, 2007; Bowen, 1978), these accounts contributed important evidence to the current debate surrounding language brokers’ roles and responsibilities.

However, there were some limitations of this study to be addressed in future research. First, while concerted efforts were made to recruit participants from a range of ethnic backgrounds, the homogeneity of the sample represents only a small proportion of Australia’s culturally and linguistically diverse population (ABS, 2017). Future research should recruit a larger, more diverse sample of participants. Secondly, the accounts provided by participants were predominately retrospective and so may have been influenced by memory bias (Colman et al., 2015). Although, retrospective accounts can be useful in gaining insight into the lasting memories of language brokering, the validity of these reports may sometimes be uncertain (Mier-Chairez et al., 2019). Additionally, demographic information related to the socio-economic background, and a timeline of brokering events would have provided valuable insight into their brokering experience. Finally, both the researchers in this study acknowledge that they are non-language brokers from CALD backgrounds. However, to overcome potential biases, the interpretation of findings was carried out with the research theme of the broader study, Language Brokers: The hidden figures.

## Conclusion

This research demonstrated that ethnic identity and traditions might act as a barrier to language brokers seeking help and support. Given that research has started to link language brokering to increased anxiety, depressive, and somatic symptoms (Arellano et al., 2018; Rainey et al., 2014), further research is warranted in this area. Another area that merits research based on the current findings is the ways in which language brokers negotiate their brokering responsibilities within the family. The factors contributing to brokers successfully or unsuccessfully negotiating their brokering responsibilities may provide important insight into additional moderating factors that promote positive outcomes for language brokers’ psychological wellbeing (Evergeti & Ryan, 2013).

Further, Australian policymakers should begin to take reasonable steps to identify the types of dedicated support language brokers and their families require. For example, the current study demonstrates the prominent role that ethnic identity plays in language brokers’ conceptualizations of their brokering experiences. In combination with past research that demonstrates the protective role of ethnic identity (Tomasi & Narchal, 2020), these findings indicate that culturally specific supports such as mentorships and ethnic community groups may be effective culturally responsive interventions (Hernandez-Plaza et al., 2005). Given that a relatively large number of the Australian migrant population is likely relying on language brokers to interpret and translate for them (ABS, 2017; Tomasi & Narchal, 2020), such supports have the potential to improve the lives and resettlement outcomes for a large group of language brokers and their families. Language brokering responsibilities compel brokers to protect, support, and care for parents in a way that constitutes a form of hidden caregiving. This is indeed a matter of concern for those interested in the wellbeing and psychological outcomes of language brokers, as care work that is invisible or hidden within the family domain is associated with an absence of support that can have profound implications for individuals performing care work (Smyth et al., 2010; Stamatopoulos, 2018).

## Data Availability

Please contact the corresponding author to access the data.
